# AIM-SEEM: Adapting SEEM for Open-Vocabulary Terrain Segmentation Across Arbitrary Imaging Modalities

**DOI:** 10.3390/s26061869

**Published:** 2026-03-16

**Authors:** Yuqian Wang, Xuefu Xiang, Yongcun Wu, Yong Zhang, Xinyue Li

**Affiliations:** 1 Southwest Automation Research Institute, Mianyang 621000, China; luoyunzhi@58suo.com (Y.W.); xxf@58suo.com (X.X.); yiranguwol@sina.cn (Y.W.); 2 School of Medical Technology, Beijing Institute of Technology, Beijing 100081, China; 3 School of Automation, Beijing Institute of Technology, Beijing 100081, China; 3220231074@bit.edu.cn

**Keywords:** terrain semantic segmentation, arbitrary imaging modalities, open-vocabulary learning, vision–text alignment

## Abstract

Terrain segmentation performance directly affects the reliability of robotic environmental perception and decision making, yet most existing methods are built upon the assumptions of fixed sensing configurations and closed label sets. As a result, they struggle to meet real world outdoor requirements where modalities can be dynamically available and semantic classes continually expand. This paper systematically studies open-vocabulary terrain segmentation under arbitrary imaging modality combinations and proposes a unified foundation model-based framework named AIM-SEEM (SEEM for Arbitrary Imaging Modalities). Built upon Segment Everything Everywhere All at Once (SEEM), AIM-SEEM performs stable input side adaptation and controlled fusion of heterogeneous modalities, maximizing the reuse of pre-trained visual priors to accommodate different modality types and counts. Furthermore, to address the distribution shifts and the resulting vision–text alignment degradation caused by modality extension, a vision-guided text calibration mechanism is introduced to preserve open-vocabulary segmentation capability under multi-modality combination inputs. Experiments on two benchmarks under three evaluation settings, including full-modality, modality-agnostic, and open-vocabulary, show that AIM-SEEM consistently outperforms prior methods.

## 1. Introduction

Terrain segmentation is a fundamental capability of robotic environmental perception because it provides dense semantic cues for scene understanding and terrain-aware representation in unstructured outdoor environments [1,2,3,4]. Its practical importance spans multiple application scenarios, including off-road autonomous driving, legged robot locomotion, planetary rover navigation, and other field robotics tasks, where reliable terrain understanding supports robust perception, environment interpretation, and subsequent high-level system reasoning under complex conditions [1,2,3,5].

Meanwhile, outdoor terrain perception increasingly relies on heterogeneous sensors because no single sensing technology can remain robust under all environmental and operational conditions. As the most widely deployed visual modality, RGB cameras provide dense appearance and texture cues at low cost, but their performance degrades under illumination variation, shadows, and adverse weather [1,4]. When explicit geometric awareness is required, LiDAR directly provides scene structure and range measurements, yet its outputs are typically sparse, relatively expensive, and less informative in fine-grained semantic texture representation [6,7]. For low-light or night-time operation, thermal infrared imaging offers more stable radiometric cues than RGB, but it often lacks detailed boundaries and rich appearance patterns [8]. In highly dynamic scenarios, event cameras respond asynchronously to brightness changes and thus provide high temporal resolution and strong robustness to rapid motion, although they contain limited static appearance information [9]. Consequently, terrain observation is shifting from single-modality RGB sensing to multi-modality perception with complementary yet dynamically available inputs [10,11,12]. At the same time, practical applications are also rapidly demanding open-vocabulary semantic understanding, where systems are no longer restricted to predefined closed class sets but must recognize and segment unseen concepts [13,14].

However, existing methods typically address only one of these requirements. To adapt to variable input modalities, Zhang et al. introduced the direction of arbitrary-modal semantic segmentation (AMSS) [12]. Many AMSS methods rely on aggressive channel expansion for modality alignment [15,16,17], which can distort modality representations and degrade accuracy. Architecturally, they often introduce complex fusion modules after independent encoding for each modality, leading to substantial increases in parameters and computation while failing to efficiently leverage the priors of pre-trained models. More importantly, although AMSS can handle modality variability, it remains constrained by closed label sets. Open-vocabulary segmentation overcomes this limitation and enables pixel-level semantic prediction from arbitrary text descriptions [18,19,20]. For RGB images, mainstream open-vocabulary segmentation methods commonly adopt a two-stage pipeline that first generates class-agnostic mask proposals and then performs classification using Contrastive Language-Image Pre-training (CLIP) [21,22,23,24]. This pipeline is prone to cascading errors, where biases from the proposal stage can propagate and amplify, ultimately degrading segmentation performance [8,25].

Motivated by these observations, the new task of open-vocabulary segmentation under arbitrary imaging modalities for terrain understanding is explicitly formulated and systematically studied. This task requires stable pixel-level semantic prediction under the dual constraints of variable modality combinations and an open class space. To this end, AIM-SEEM is proposed on the basis of SEEM [26]. ROA and CMB are introduced to enable stable integration of arbitrary modality combinations while keeping the backbone largely unchanged, thereby maximizing the reuse of its pre-trained visual priors. However, modality extension introduces significant distribution shifts that disrupt the original vision–text alignment in SEEM, causing its open-vocabulary capability to deteriorate noticeably under non-RGB inputs. To address this alignment degradation induced by modality extension, the Visual-Guided Text Tuner (VGTT) is introduced to perform vision-guided adaptive calibration of text representations, thereby restoring robust open-vocabulary segmentation under arbitrary modalities. The main contributions of this work are:

- The new task of open-vocabulary terrain segmentation under arbitrary imaging modalities is proposed and systematically studied, which unifies the key challenges introduced by variable modality combinations and an open class space.
- AIM-SEEM is presented as a SEEM based framework that supports stable integration of arbitrary modality combinations while maximizing the reuse of pre-trained visual priors. Vision guided adaptive text calibration is further introduced to alleviate vision–text alignment degradation caused by cross modality distribution shifts.
- Comprehensive evaluations are conducted on two terrain segmentation benchmarks under three experimental settings, including full-modality, modality-agnostic, and open-vocabulary. The results show consistent gains in accuracy, robustness, and generalization, and AIM-SEEM outperforms prior methods.

The remainder of this paper is organized as follows. Section 2 reviews related studies on terrain segmentation, arbitrary-modality semantic segmentation, and open-vocabulary segmentation. Section 3 presents the proposed AIM-SEEM framework, including the overall architecture and the designs of ROA, CMB, and VGTT. Section 4 describes the datasets, experimental settings, and experimental results under the full-modality, modality-agnostic, and open-vocabulary settings. Section 5 concludes this paper and discusses future research directions.

## 2. Related Works

### 2.1. Terrain Segmentation

Terrain segmentation provides dense semantic priors for ground robots to assess traversability and risk in unstructured outdoor environments. Most existing terrain segmentation methods are built upon two common assumptions. The first assumption is a fixed sensing configuration. Some methods focus on RGB only perception and train and deploy a semantic segmentation backbone under a single RGB camera setup [5]. Other studies improve robustness by fusing complementary sensors under a fixed modality pair. Forkel et al. [6] and Lu et al. [7] investigate camera and LiDAR fusion, integrating geometric cues from LiDAR with image features to enhance recognition under illumination changes and ambiguous textures. Huang et al. [11] further incorporate inertial information as auxiliary features and explore RGB and inertial measurement unit fusion to improve perception stability under high speed motion and vibration. Although these methods work well under their target configurations, the learned representations are typically strongly coupled with the assumed modalities. When sensor failures occur or platform dependent sensing setups differ so that the available modalities change, performance often degrades significantly. The second assumption is a closed label set. Many terrain segmentation methods follow the closed set supervised paradigm of semantic segmentation, where the prediction space and the training objective are tied to a predefined set of terrain classes. A typical practice is to train pixel level classifiers under a specific dataset or application label system, such as was performed by Moore et al. [27] and Wen et al. [28], which learn decision boundaries over fixed classes defined by their annotation protocols. Since the output class space is fixed during training, when deployment encounters terrain concepts outside the predefined label system, the model often assigns them to the closest seen class or to background. This leads to semantic confusion and reduces the reliability of downstream risk assessment and decision making.

Different from prior studies based on fixed modalities or closed label sets, this paper investigates open-vocabulary terrain segmentation under arbitrary imaging modality combinations. It requires jointly addressing two core challenges, variable modality combinations and an open class space.

### 2.2. Arbitrary Modality Semantic Segmentation

Arbitrary modality semantic segmentation (AMSS) aims to maintain consistent predictions when the type and the number of input modalities vary at inference time [12]. A representative line of research designs modality specific encoders to extract features for each modality and then aggregates complementary information through cross modality fusion modules. CMNeXt [12] follows a modality specific encoding and cross modality fusion framework, treating one modality as the primary representation and the remaining modalities as complementary cues. It employs selective fusion so that a stable unified feature can be formed under missing modalities or changing modality combinations. MAGIC [15] considers that modality reliability can vary with the scene and derives a modality agnostic aggregated representation as a reference. It then adaptively selects and weights more reliable modality features to improve robustness under sensor failures and distribution discrepancies. MMSFormer [16] uses transformer based modality specific encoders to obtain hierarchical features and performs unified multi-scale fusion with channel re calibration. This design supports diverse modality combinations within a single architecture and yields consistent predictions. U3M [17] emphasizes avoiding modality bias by modeling all modalities in an equal manner and continuously fusing multi-scale modality information into a unified representation, which helps maintain stable segmentation when the dominant modality changes or some modalities are missing. To satisfy dimensional consistency, many AMSS methods rely on direct alignment operations such as channel expansion or feature concatenation, which often alter modality statistics and introduce distribution shifts. They also introduce heavy interaction modeling through complex fusion modules such as multi-scale attention, which limits efficient reuse of pre-trained model priors.

In contrast, this paper adopts an input side adaptation strategy and introduces a dedicated modality alignment module that maps heterogeneous modalities into a representation space that is reusable by a foundation model. This enables stable integration of arbitrary modality combinations with minimal backbone modification and allows more effective reuse of the foundation model pre-trained capability.

### 2.3. Open-Vocabulary Segmentation

Open-vocabulary segmentation (OVS) leverages the semantic transfer capability of vision–text pre-training to recognize classes beyond the training label set [18,19,20,29]. Most existing OVS methods are designed for RGB only inputs, and only a small number of studies explore fixed non-RGB modality settings, such as RGB and thermal imaging [8] or event camera inputs [9]. Early works [21,30,31,32] commonly follow a two stage paradigm that first generates class agnostic mask proposals and then performs open-vocabulary classification using CLIP [33]. With the emergence of segmentation foundation models such as the Segment Anything Model (SAM) [34], recent studies further use SAM to improve the quality of mask proposals, while the overall pipeline still decouples proposal generation from semantic assignment [22,23,24,25]. This two stage pipeline is highly sensitive to proposal quality, since misses and boundary errors in the proposal stage can propagate to the semantic assignment stage and cause cascading errors, which limits the upper bound of final segmentation performance.

Different from prior work, this paper does not focus on improving the two-stage open-vocabulary segmentation paradigm. Starting from SEEM [26], which already supports open-vocabulary segmentation under RGB inputs, vision-guided text calibration is introduced to alleviate alignment degradation induced by modality shift, thus maintaining reliable open-vocabulary segmentation under multi-modal combinations.

## 3. Materials and Methods

AIM-SEEM is an open-vocabulary terrain segmentation architecture developed by extending SEEM to support arbitrary imaging modalities. Section 3.1 outlines the overall framework. Section 3.2, Section 3.3 and Section 3.4 then detail its three core components: the RGB-Oriented Aligner (ROA), the Cross-Modal Blender (CMB), and the Visual-Guided Text Tuner (VGTT).

### 3.1. AIM-SEEM Architecture

**Figure 1:**
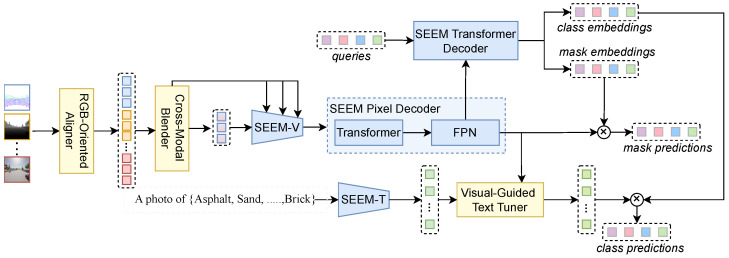
Architecture of AIM-SEEM. Newly introduced customized modules are highlighted in yellow-filled blocks.

Figure 1 illustrates the architecture of AIM-SEEM. The design targets a practical gap when extending RGB-grounded open-vocabulary segmentation to arbitrary imaging modalities: modality shift and modality combinations can degrade the usability of RGB-pretrained priors and weaken vision–text alignment, even if the overall backbone remains unchanged. To address this, three customized modules are introduced around SEEM while keeping its core encoders intact, so that open-vocabulary recognition and segmentation remain stable under non-RGB inputs and heterogeneous modality subsets.

Built upon SEEM’s basic framework, AIM-SEEM retains the original visual encoder (SEEM-V) and text encoder (SEEM-T). In the visual stream, ROA and CMB handle heterogeneous channel dimensions and varying modality combinations by aligning modality-specific inputs into an RGB-compatible representation space and performing controlled cross-modal blending across multiple resolutions. In the text stream, VGTT refines text embeddings using modality-specific visual cues, which improves vision–text alignment under modality shift and is critical for open-vocabulary prediction beyond RGB.

Formally, let M={1,…,M} denote the modality index set, where M=|M| is the number of modalities. For each m∈M, the input is $I^{\left(m\right)}\in R^{H\times W\times C^{\left(m\right)}}$, where $C^{\left(m\right)}$ may vary across modalities. ROA aligns each modality to a 4× feature map. CMB then blends these aligned features to produce a unified multi-resolution representation $\left\{z_{0}^{4\times },z_{0}^{8\times },z_{0}^{16\times },z_{0}^{32\times }\right\}$, which is fed into the four hierarchical stages $\left\{F_{0},F_{1},F_{2},F_{3}\right\}$ of SEEM-V.

In Stage 1, the blended 4× feature is processed as follows:

(1) $$z^{4\times }=F_{0}\;\left(z_{0}^{4\times }\right).$$

**Figure 2:**
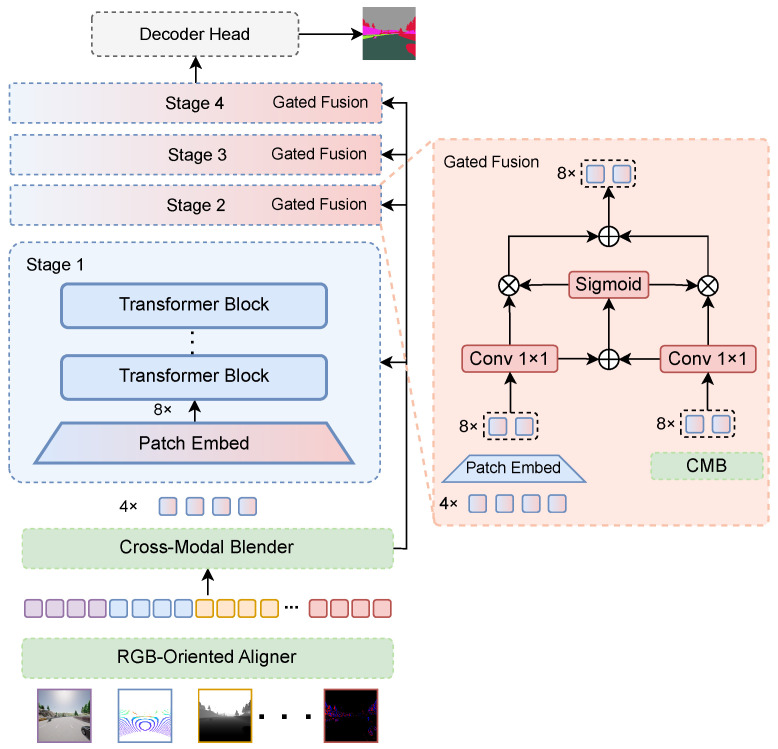
Gated fusion module.

For subsequent stages k∈{1,2,3} with resolutions $s_{k}\in \left\{8\times ,16\times ,32\times \right\}$, let $s_{0}=4\times$. The self-encoded feature $u^{s_{k}}=F_{k}\;\left(z^{s_{k-1}}\right)$ is first computed. This is then fused with the corresponding CMB-blended feature $v^{s_{k}}=z_{0}^{s_{k}}$ via a gated convex combination (see Figure 2):

(2) $$z^{s_{k}}=G^{s_{k}}⊙u^{s_{k}}+\left(1-G^{s_{k}}\right)⊙v^{s_{k}},$$

where ⊙ is element-wise multiplication. The gating map $G^{s_{k}}$ is computed from both feature branches:

(3) $$G^{s_{k}}=\sigma \left(\phi_{1}\left(u^{s_{k}}\right)+\phi_{2}\left(v^{s_{k}}\right)\right),$$

where σ is the sigmoid function and $\phi_{1},\phi_{2}$ are learnable 1×1 convolutional layers. The final multi-scale visual features are $\left\{z^{4\times },z^{8\times },z^{16\times },z^{32\times }\right\}$.

These features are upsampled by the pixel decoder to obtain per-pixel embeddings $\varepsilon_{\mathrm{pixel}}$. Concurrently, the Transformer decoder uses *Q* learnable queries to generate class embeddings $\varepsilon_{\mathrm{class}}$ and mask embeddings $\varepsilon_{\mathrm{mask}}$. Binary masks are predicted via the dot product between $\varepsilon_{\mathrm{pixel}}$ and $\varepsilon_{\mathrm{mask}}$.

For class names $\left\{C_{1},\ldots ,C_{N}\right\}$, descriptive sentences are generated using a predefined template and encoded by SEEM-T, yielding initial text embeddings $\left\{T_{1},\ldots ,T_{N}\right\}$. VGTT refines these into $\left\{T_{1}^{\prime },\ldots ,T_{N}^{\prime }\right\}$ by incorporating modality-specific visual evidence. Class prediction is performed by matching the refined text embeddings with $\varepsilon_{\mathrm{class}}$.

### 3.2. RGB-Oriented Aligner

**Figure 3:**
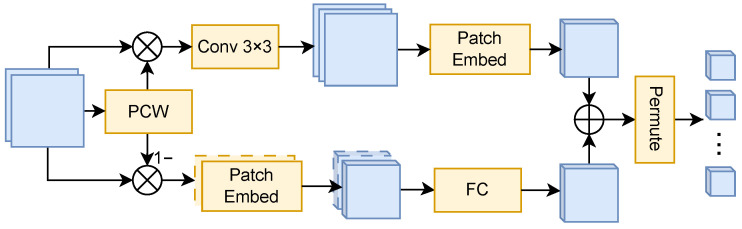
RGB-Oriented Aligner (ROA).

The RGB-Oriented Aligner (ROA) mitigates modality misalignment between arbitrary target modalities and the RGB-pretrained backbone by converting heterogeneous inputs into an RGB-friendly token sequence. Different from alignment strategies that rely on heavy modality-specific encoders or channel expansion, ROA performs an input side conversion to an RGB-compatible representation with minimal changes to the RGB-pretrained backbone, thereby maximizing the reuse of pretrained visual priors under non-RGB inputs. As shown in Figure 3, given an input modality $I\in R^{H\times W\times K}$, ROA produces a unified aligned representation for subsequent cross-modal processing.

ROA first predicts a scalar adaptive weight w∈(0,1) via Pyramid Channel Weighting (PCW), whose structure is included in Figure 3. Formally, PCW is defined as a composition of four operators (UpConv, LayerNorm, ReLU, DownConv) followed by average pooling:

(4) $$w=\sigma \left(\mathrm{AvgPool}\left(\mathrm{DownConv}(\mathrm{ReLU}(\mathrm{LN}(\mathrm{UpConv}(I))))\right)\right),$$

where LN(·) denotes LayerNorm, and σ(·) denotes the sigmoid function, ensuring w∈(0,1). The resulting *w* is used to construct two complementary reweighted inputs:

(5) $$I_{\det}=w\;I,\;I_{\mathrm{ctx}}=\left(1-w\right)\;I.$$

Here, $I_{\det}$ is intended to emphasize informative local structures (e.g., boundaries and fine patterns), whereas $I_{\mathrm{ctx}}$ is designed to retain stable contextual cues (e.g., large homogeneous regions), yielding complementary modal adaptation without relying on any explicit frequency-domain assumptions.

Next, as depicted in Figure 3, the detail-enhancement branch expands channels via a 3×3 convolution and applies patch embedding with 4× downsampling to obtain $I_{\det}^{4\times }$. In parallel, the context-preservation branch applies channel-wise patch embedding followed by a lightweight fully-connected projection to match the channel dimension, producing $I_{\mathrm{ctx}}^{4\times }$. The two branches are fused by element-wise summation:

(6) $$I^{4\times }=I_{\det}^{4\times }+I_{\mathrm{ctx}}^{4\times }.$$

Finally, ROA permutes the fused feature map into a token sequence as the aligned output:

(7) $$x^{4\times }=\Pi \;\left(I^{4\times }\right)\in R^{\left(\frac{H}{4}\frac{W}{4}\right)\times C},$$

where Π(·) denotes flattening the spatial grid into tokens.

### 3.3. Cross-Modal Blender

**Figure 4:**
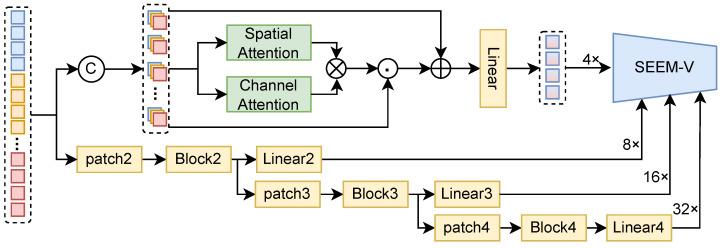
Cross-Modal Blender (CMB).

The Cross-Modal Blender (CMB) extends the segmentation capability of the RGB-pretrained backbone to arbitrary modality combinations by enabling cross-modal interaction and adaptive fusion. The novelty of CMB lies in controlled multi-resolution blending, where complementary cues are integrated only when reliable and modality-specific noise is suppressed via gated fusion, which improves robustness to missing or unreliable sensors and prevents performance collapse under arbitrary modality subsets. As shown in Figure 4, CMB takes the multi-modal 4× aligned features produced by ROA as input and generates multi-resolution blended features $\left\{z_{0}^{4\times },z_{0}^{8\times },z_{0}^{16\times },z_{0}^{32\times }\right\}$.

Cross-modal concatenation. For a given sample, the token sequence $x^{4\times ,(m)}$ of each modality is reshaped back to a spatial grid $X^{4\times ,(m)}\in R^{H_{4}\times W_{4}\times C}$, with $\left(H_{4},W_{4}\right)=\left(H/4,W/4\right)$. All modalities are then concatenated along the channel dimension:
  (8) $$X^{4\times }=\mathrm{Concat}{\left(X^{4\times ,(m)}\right)}_{m\in M}\in R^{H_{4}\times W_{4}\times \left(MC\right)}.$$
Spatial-channel attention blending. CMB applies a spatial-channel attention mechanism to adaptively reweight multimodal features. The spatial attention map $A_{s}\in R^{H_{4}\times W_{4}\times 1}$ and the channel attention vector $A_{c}\in R^{1\times 1\times (MC)}$ are defined as
  (9) $$A_{s}=\sigma \left(\mathrm{Conv}\left([{\mathrm{AvgPool}}_{c}\left(X^{4\times }\right);{\mathrm{MaxPool}}_{c}\left(X^{4\times }\right)]\right)\right),$$
  (10) $$A_{c}=\sigma \left(\mathrm{MLP}\left({\mathrm{AvgPool}}_{hw}\left(X^{4\times }\right)\right)+\mathrm{MLP}\left({\mathrm{MaxPool}}_{hw}\left(X^{4\times }\right)\right)\right),$$
  where ${\mathrm{AvgPool}}_{c}/{\mathrm{MaxPool}}_{c}$ pool along the channel dimension to produce a spatial map, and ${\mathrm{AvgPool}}_{hw}/{\mathrm{MaxPool}}_{hw}$ pool over spatial dimensions to produce a channel descriptor. Using element-wise multiplication with broadcasting, the blended 4× feature is computed as
  (11) $$Z_{0}^{4\times }=\mathrm{Linear}\left(X^{4\times }+\left(A_{s}⊙A_{c}⊙X^{4\times }\right)\right),$$
  where ⊙ denotes element-wise multiplication with broadcasting. Finally, $Z_{0}^{4\times }$ is flattened into tokens to match the SEEM-V input format:
  (12) $$z_{0}^{4\times }=\Pi \;\left(Z_{0}^{4\times }\right).$$
Multi-resolution generation. To provide multi-scale cues for segmentation, CMB generates lower-resolution blended features in parallel. For each target resolution s∈{8×,16×,32×}, a patch embedding operator ${\mathrm{Patch}}_{s}\left(\cdot \right)$ is applied to each modality, followed by cross-modal aggregation and refinement:
  (13) $${\tilde{X}}^{s}=\mathrm{Concat}{\left({\mathrm{Patch}}_{s}\left(X^{4\times ,(m)}\right)\right)}_{m\in M}\in R^{H_{s}\times W_{s}\times C_{s}},$$
  (14) $$Z_{0}^{s}=\mathrm{Linear}\left(\mathrm{Block}({\tilde{X}}^{s})\right),\;z_{0}^{s}=\Pi \;\left(Z_{0}^{s}\right),$$
  where $\left(H_{s},W_{s}\right)=\left(H/s,W/s\right)$, and Block(·) denotes a lightweight feature mixing block (e.g., a Transformer/multi-layer perceptron (MLP) block) operating at the corresponding resolution. This parallel design allows the model to exploit multi-resolution information while maintaining consistent fusion behavior across modality combinations.

### 3.4. Visual-Guided Text Tuner

When the visual stream is extended from RGB to non-RGB modalities, the distribution of pixel-level features may deviate from the alignment space learned by the RGB-grounded vision–language model, which directly harms open-vocabulary recognition even if the segmentation backbone remains strong. VGTT addresses this previously under-discussed failure mode by refining text tokens with modality-specific visual evidence from the pixel decoder, so that the resulting text representations better match the current visual feature distribution.

Let $T_{0}\in R^{N\times D}$ denote the text embeddings of *N* class prompts produced by SEEM-T, where *N* is the number of class prompts (i.e., classes) and *D* is the embedding dimension. Let $\varepsilon_{\mathrm{pixel}}\in R^{H\times W\times C}$ denote the pixel-decoder output. It is reshaped into a token sequence $P\in R^{N_{p}\times C}$, with $N_{p}=HW$, and projected to the same embedding dimension via a learnable linear layer $W_{p}\in R^{C\times D}$.

(15) $$V^{0}=PW_{p},\;V^{0}\in R^{N_{p}\times D}.$$

In VGTT, the visual tokens serve as a fixed memory for cross-attention, i.e., $V^{l}\equiv V^{0}$ for all layers *l*.

VGTT consists of *L* stacked cross-attention Transformer layers, with *L* set to 2. Each layer adopts a pre-normalization design and contains (i) a multi-head cross-attention (CA) sublayer and (ii) a position-wise feed-forward network (FFN) sublayer, both equipped with residual connections. For the *l*-th layer (l=0,1,…,L−1), layer normalization is first applied:

(16) $${\hat{T}}^{l}=\mathrm{LN}\left(T^{l}\right),\;{\hat{V}}^{l}=\mathrm{LN}\left(V^{0}\right).$$

The multi-head cross-attention is defined as

(17) $$\mathrm{CA}\left({\hat{T}}^{l},{\hat{V}}^{l}\right)=\mathrm{Concat}\left({\mathrm{head}}_{1},\ldots ,{\mathrm{head}}_{h}\right)W_{o},$$

(18) $${\mathrm{head}}_{j}=\mathrm{Softmax}\;\left(\frac{\left({\hat{T}}^{l}W_{q}^{j}\right){\left({\hat{V}}^{l}W_{k}^{j}\right)}^{⊤}}{\sqrt{d}}\right)\left({\hat{V}}^{l}W_{v}^{j}\right),$$

where *h* is the number of attention heads, d=D/h, and $W_{q}^{j},W_{k}^{j},W_{v}^{j}\in R^{D\times d}$ and $W_{o}\in R^{D\times D}$ are learnable projection matrices. The text tokens are then updated via residual connections:

(19) $${\tilde{T}}^{l}=T^{l}+\mathrm{CA}\left({\hat{T}}^{l},{\hat{V}}^{l}\right),$$

(20) $$T^{l+1}={\tilde{T}}^{l}+\mathrm{FFN}\left(\mathrm{LN}({\tilde{T}}^{l})\right).$$

The feed-forward network FFN(·) is implemented as a two-layer MLP with a non-linear activation. This design enables each text token to attend to modality-specific pixel evidence through cross-attention, while preserving the original semantics through pre-normalization and residual learning.

After *L* layers, refined text embeddings $T^{L}\in R^{N\times D}$ are obtained. These refined text representations are then used in the subsequent vision–text matching and mask decoding procedures following SEEM.

Overall, AIM-SEEM is not a simple modality-fusion variant of SEEM. The contribution is to jointly preserve pretrained visual priors and language grounding under modality shift, by aligning heterogeneous inputs, blending modalities in a controlled manner across resolutions, and re-calibrating text embeddings with modality-conditioned visual evidence. These components form a closed loop that maintains open-vocabulary segmentation performance beyond RGB and under arbitrary modality combinations.

## 4. Experiments

### 4.1. Experimental Setup

#### 4.1.1. Datasets

Due to the lack of widely used multimodal benchmarks for terrain segmentation, two datasets that provide aligned multimodal observations and fine-grained terrain annotations are used. (i) The MCubes dataset [35] provides four aligned modalities, including RGB, near-infrared (NIR), degree of linear polarization (DoLP), and angle of linear polarization (AoLP). Nine terrain-related classes are selected, including Asphalt, Concrete, Road Marking, Sand, Gravel, Cobblestone, Brick, Grass, and Water. (ii) The DELIVER dataset [12] provides four paired modalities, including RGB, depth, LiDAR, and event. Eight terrain-related classes are selected, including Road Line, Road, SideWalk, Vegetation, Bridge, Rail Track, Ground Rail, and Water. Both datasets provide aligned multi-modality imaging inputs that can be directly fed into the model as multi-channel inputs without additional preprocessing.

#### 4.1.2. Experimental Settings and Evaluation Metrics

The proposed method is evaluated under three settings: full-modality, modality-agnostic, and open-vocabulary, which assess multi-modal fusion capability, robustness to arbitrary modality inputs, and generalization to unseen terrain classes, respectively.

Full-modality setting. This setting evaluates the model’s capability to fuse multiple modalities by using all available modalities during both training and testing. The class-wise Intersection over Union (IoU) and the mean IoU (mIoU) are reported. For a class *c*, IoU is defined as

(21) $${\mathrm{IoU}}_{c}=\frac{{\mathrm{TP}}_{c}}{{\mathrm{TP}}_{c}+{\mathrm{FP}}_{c}+{\mathrm{FN}}_{c}},$$

and the mean IoU over *C* classes is computed as

(22) $$\mathrm{mIoU}=\frac{1}{C}\sum_{c=1}^{C}{\mathrm{IoU}}_{c}.$$

Modality-agnostic setting. This setting evaluates the model’s robustness under arbitrary modality inputs. The model is trained with all modalities and tested with any modality combination. For each modality combination s∈S, ${\mathrm{mIoU}}^{\left(s\right)}$ is computed, and the averaged performance across all combinations is additionally reported:

(23) $${\mathrm{mIoU}}_{\mathrm{avg}}=\frac{1}{\left|S\right|}\sum_{s\in S}{\mathrm{mIoU}}^{\left(s\right)}.$$

Open-vocabulary setting. This setting evaluates the generalization ability of the model to terrain classes that are not included in the predefined fixed vocabulary. Following the common protocol (e.g., KgCoOp [36]), the label space is split into base classes and new classes. The model is primarily trained on base classes and evaluated on both subsets simultaneously. ${\mathrm{mIoU}}_{\mathrm{base}}$ and ${\mathrm{mIoU}}_{\mathrm{new}}$ are reported. Importantly, because open-vocabulary segmentation must balance performance on both seen and unseen classes, the primary metric is the harmonic mean IoU (hIoU):

(24) $$\mathrm{hIoU}=\frac{2\;{\mathrm{mIoU}}_{\mathrm{base}}\cdot {\mathrm{mIoU}}_{\mathrm{new}}}{{\mathrm{mIoU}}_{\mathrm{base}}+{\mathrm{mIoU}}_{\mathrm{new}}}.$$

#### 4.1.3. Implementation Details

The SEEM V1 model [26] is adopted under its original configuration, retaining the 101 class-agnostic mask proposals. The model is optimized with AdamW (weight decay 0.05), using a learning rate of $1\times 10^{-5}$ for most modules, except for the SEEM-V and SEEM-T components, which use $1\times 10^{-6}$. During training, all input images are randomly cropped to 512×512 pixels. Experiments are conducted on 8 NVIDIA GeForce RTX 3090 GPUs (NVIDIA Corporation, Santa Clara, CA, USA), and each model is trained for 300 epochs.

### 4.2. Main Results

The proposed method is compared with state-of-the-art arbitrary-modality segmentation models, including CMNeXt [12], MMSFormer [16], Magic [15], and U3M [17], all using SegFormer-B2 [37] as the backbone.

#### 4.2.1. Full-Modality Evaluation

Under the full-modality setting, the proposed approach demonstrates clear advantages over existing methods. Specifically, the proposed method improves mIoU by 7.88 and 5.03 percentage points on the two datasets, respectively, and achieves the best results for most classes, as shown in Table 1. On MCubes, the proposed method performs particularly well on the Gravel and Water classes. The previous best method reports an mIoU of 68.5% on Gravel, whereas the proposed method increases it to 84.3%, yielding a gain of 15.8 percentage points. For Water, the prior best mIoU is 61.3%, and the proposed method improves it to 75.4%, corresponding to a gain of 14.1 percentage points. On DELIVER [12], the proposed method achieves the best or second-best performance across all classes. The largest gain is observed on Rail Track, where the previous best mIoU is 63.4% and the proposed method raises it to 80.5%, improving by 17.1 percentage points. These results indicate that the proposed model effectively leverages multi-modal information to enhance segmentation accuracy.

#### 4.2.2. Modality-Agnostic Evaluation

Under the modality-agnostic setting, Table 2 and Table 3 report the performance under arbitrary modality combinations on MCubes and DELIVER, respectively. The proposed method achieves the best mean performance on both datasets, reaching an mIoU of 39.15% on MCubes and an mIoU of 43.76% on DELIVER, which corresponds to mean mIoU gains of 0.41 and 6.83 percentage points over the strongest baseline.

**Figure 5:**
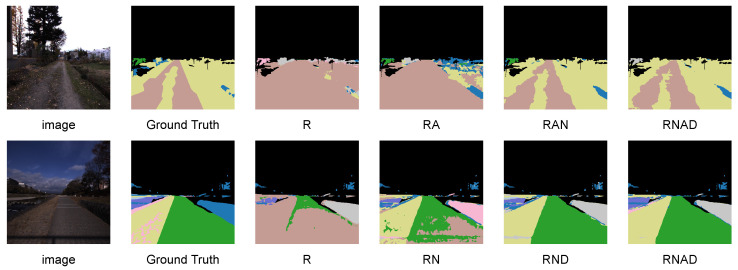
Visualization of arbitrary inputs using RGB, AoLP, DoLP, and NIR on MCubes. The first column shows input images, followed by ground truth and predicted semantic maps under different modality combinations. All colored maps denote semantic segmentation results, where each color corresponds to a terrain category.

On MCubes, AIM-SEEM remains highly effective when RGB is absent. Using only the NIR modality, an mIoU of 28.44% is attained, improving upon the best competing result by 25.14 percentage points. With the three-modality combination excluding RGB, namely AoLP-DoLP-NIR (ADN), an mIoU of 41.22% is further achieved, exceeding the best baseline by 3.65 percentage points. These results indicate that the proposed adaptation generalizes well to non-RGB modalities and sustains strong performance under input modality variations. As shown in Figure 5, qualitative visualizations are provided under representative modality combinations on MCubes.

**Figure 6:**
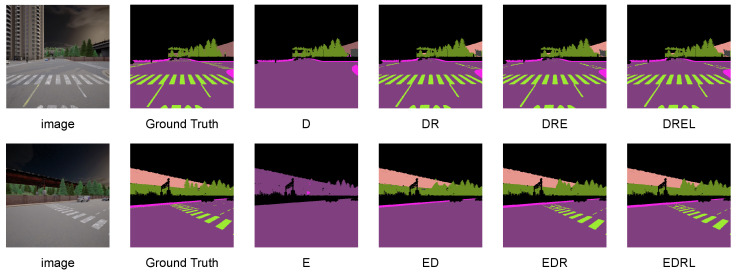
Visualization of arbitrary inputs using RGB, Depth, Event, LiDAR on DELIVER. The first column shows input images, followed by ground truth and predicted semantic maps under different modality combinations. All colored maps denote semantic segmentation results, where each color corresponds to a terrain category.

On DELIVER, the advantage is more pronounced. AIM-SEEM attains the best or second best performance for the vast majority of modality combinations and yields clear improvements in challenging non-RGB cases. In particular, it achieves an mIoU of 9.25% with only Event and an mIoU of 9.24% with only LiDAR, outperforming the best competing methods by 7.88 and 5.14 percentage points, respectively. When using the Event and LiDAR combination, it reaches an mIoU of 9.25%, improving by 5.40 percentage points. Overall, these results demonstrate that AIM-SEEM is robust to arbitrary modality inputs and substantially reduces reliance on RGB by strengthening representation and fusion for non-RGB modalities. As shown in Figure 6, qualitative visualizations are provided under representative modality combinations on DELIVER.

#### 4.2.3. Open-Vocabulary Evaluation

Under the open-vocabulary setting, Table 4 compares different methods on MCubes and DELIVER with 1/4/8 labeled samples per new class. The central challenge of open-vocabulary segmentation is to generalize to new classes under extremely limited supervision while maintaining strong performance on base classes. hIoU, namely the harmonic mean of base-class and new-class mIoU, is therefore adopted as the primary metric because it directly reflects the balance between the two and discourages methods that excel only on one side.

Across all shot settings, AIM-SEEM achieves the best overall balance. On MCubes and DELIVER, it improves the mean hIoU by 8.28 and 7.79 percentage points, respectively, compared with the strongest baseline. The improvement is mainly driven by consistently higher mIoU on new classes across different shots, whereas the mIoU on base classes shows only a small decrease, around two percentage points on average. This trend is consistent with the goal of open-vocabulary learning, where the key requirement is reliable recognition of previously unseen classes beyond the training label set. Overall, the consistently higher hIoU indicates that the proposed method achieves a more favorable balance between base and new classes while delivering substantially stronger generalization to new classes.

### 4.3. Ablation Study

#### 4.3.1. Component-Wise Ablations

To clarify the contribution of each component in AIM-SEEM, SEEM [26] fine-tuned on RGB data is adopted as the baseline, and modules are progressively added under the full-modality setting on MCubes, as reported in Table 5. The RGB-only baseline achieves an mIoU of 44.32%, indicating that relying on a single modality is insufficient in this setting. Introducing single-scale CMB for four-modality fusion raises the performance to an mIoU of 65.86%, corresponding to a gain of 21.54 percentage points, which shows that CMB effectively extracts modality-specific cues and performs an initial integration that compensates for the information bottleneck of RGB. Multi-scale CMB is further examined by combining its fused features with the SEEM visual branch features. Direct summation leads to training collapse, and simple averaging reduces performance to an mIoU of 54.25%. In contrast, gated fusion yields an mIoU of 67.85%, improving over the single-scale variant by 1.99 percentage points, which suggests that dynamic weighting is crucial for suppressing noisy or redundant modality features during fusion. Adding ROA further improves performance to an mIoU of 71.09%, a gain of 3.24 percentage points, because it avoids forced channel expansion for non-RGB modalities and better preserves modality-specific feature distributions. Finally, incorporating VGTT boosts performance to an mIoU of 73.13%, adding 2.04 percentage points, which indicates that jointly optimizing the visual and text branches enhances cross-modal semantic consistency and strengthens class understanding under multi-modal inputs.

#### 4.3.2. Base-New Trade-Off

In the open-vocabulary setting, performance on base classes and previously unseen new classes is reported together with the harmonic mean *H* to reflect the balance between them under the same 8-shot protocol. As shown in Table 6, introducing VGTT consistently improves both recognition strength and the base-new balance. Specifically, the base score increases from 60.38% to 65.05%, while the new score increases from 41.03% to 42.96%. As a result, *H* rises from 48.86% to 51.75%, corresponding to a gain of 2.89 percentage points. These results suggest that the proposed text calibration helps preserve vision–text alignment under modality shift and improves overall open-vocabulary transfer.

**Figure 7:**
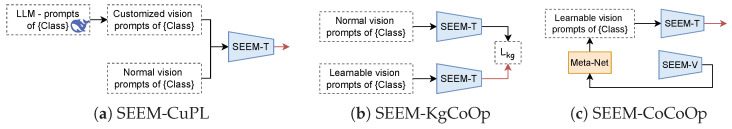
Base-new trade-off strategy. The red arrow indicates the final output of SEEM-T, while the other arrows only denote the general information flow.

Building on the VGTT baseline, the potential of representative prompt-based strategies to improve the base-new balance is further investigated. As illustrated in Figure 7a, CuPL [38] is adapted to SEEM by enriching class text prompts without additional training, while the SEEM mask prediction pipeline is kept unchanged and the final SEEM-T output is evaluated. The normal prompts follow the original SEEM setting, whereas the customized prompts are generated by querying DeepSeek R1 [39] with the template in Table 7. Table 6 shows that CuPL slightly improves the new score but reduces the base score, leading to a marginal decrease of *H* from 51.75% to 51.61%.

Two learnable prompt-tuning methods are also integrated, as shown in Figure 7b,c. KgCoOp [36] learns continuous context tokens with a knowledge-guided constraint to stabilize transfer, while CoCoOp [40] introduces an image-conditioned prompt generator for instance-adaptive prompts at inference time. As reported in Table 6, both methods improve the harmonic mean over the VGTT baseline, with KgCoOp reaching an *H* of 52.16% and CoCoOp reaching an *H* of 52.15%, indicating a more favorable base-new balance under the same 8-shot setting.

## 5. Conclusions and Future Work

AIM-SEEM is proposed as a unified framework for open-vocabulary terrain segmentation under arbitrary imaging modalities and their combinations. The main contribution lies in formulating and addressing the open-vocabulary arbitrary-modality terrain segmentation problem through a unified pipeline that remains effective when the sensing modality changes and when multiple modalities are combined. To this end, three tightly coupled components are integrated into a coherent adaptation, fusion, and calibration process that jointly bridges the modality gap and preserves language grounding. Specifically, ROA converts heterogeneous modality features into an RGB-compatible representation space, which enables stable reuse of RGB-pretrained vision priors under non-RGB inputs by reducing feature distribution mismatch. Based on the aligned features, CMB performs controlled cross-modal blending and generates multi-resolution representations consistent with the hierarchical inputs expected by the vision encoder, while gated fusion selectively integrates complementary cues and down-weights modality-specific noise, preventing performance degradation when some sensors are unreliable. With a more stable visual feature distribution, VGTT further calibrates class text embeddings using modality-specific pixel evidence from the decoder, compensating for modality-induced vision–text misalignment and maintaining consistent language grounding for open-vocabulary recognition. With these designs, AIM-SEEM achieves state-of-the-art performance across three experimental setups on two benchmark datasets and shows clear advantages on non-RGB modalities.

Although the results demonstrate strong capability in handling arbitrary modalities, there remains room to improve absolute accuracy and stability when RGB is unavailable. Future work will focus on strengthening fault tolerance, including more reliable modality selection and uncertainty-aware fusion for alternative modalities. In addition, since the present study primarily emphasizes segmentation effectiveness, modality adaptability, and open-vocabulary generalization, efficiency-oriented optimization remains outside the main scope of this work. Therefore, computational cost control, model compression, and deployment acceleration will also be important directions for future research so as to improve practical applicability on resource-constrained platforms. The trade-off between base-class performance and novel-class generalization in open-vocabulary transfer also warrants further investigation. This trade-off is not introduced by AIM-SEEM but becomes more apparent when the model is pushed toward stronger open-vocabulary generalization. Ablation results with prompt-based strategies suggest that the balance can be improved to some extent, yet it is not fully resolved, motivating more principled mechanisms for balancing base and novel classes in open-vocabulary terrain segmentation.

## Figures and Tables

**Table 1:** IoU (%) in the full-modality setting on the MCubes and DELIVER datasets. Bold indicates the best, and underline indicates the second-best performance.

**MCubes**										
Methods	Asp.	Conc	R.M.	Sand	Gravel	Cobb.	Brick	Grass	Water	Mean
CMNeXt [12]	84.7	45.2	74.8	67.8	67.1	68.7	43.2	58.9	54.4	62.76
MMSFormer [16]	86.5	61.8	70.5	63.7	68.4	66.8	45.8	**77.2**	46.6	65.25
U3M [17]	84.2	42.9	71.1	**73.3**	68.5	73.4	43.6	56.3	61.3	63.84
Magic [15]	88.9	50.8	**75.0**	66.9	66.8	71.6	46.5	55.3	54.7	64.05
AIM-SEEM	**90.7**	**65.1**	70.0	72.7	**84.3**	**75.2**	**52.6**	72.2	**75.4**	**73.13**
**DELIVER**										
Methods	R.L.	Road	S.W.	Veget.	Bridge	R.T.	G.R.	Water		Mean
CMNeXt [12]	**85.9**	98.2	82.3	89.0	53.6	61.5	55.0	24.0		68.69
MMSFormer [16]	80.0	97.1	72.8	89.7	42.1	20.7	27.4	52.0		60.23
U3M [17]	82.9	97.8	57.0	84.7	68.8	63.4	10.7	32.8		62.26
Magic [15]	84.8	98.4	86.2	88.7	59.3	59.8	**73.1**	30.9		72.65
AIM-SEEM	85.6	**99.2**	**92.4**	**99.4**	**77.2**	**80.5**	31.7	**55.4**		**77.68**

**Table 2:** mIoU (%) in the modality-agnostic setting on the MCubes dataset. R denotes RGB, A denotes angle of linear polarization (AoLP), D denotes degree of linear polarization (DoLP), and N denotes near-infrared (NIR). Multi-letter entries indicate modality combinations, such as RA for RGB plus AoLP and RADN for using all four modalities. Bold indicates the best, and underline indicates the second-best performance.

**Methods**	**R**	**A**	**D**	**N**	**RA**	**RD**	**RN**	**AD**
CMNeXt [12]	1.86	1.54	2.51	2.28	47.96	43.67	45.90	6.99
MMSFormer [16]	**64.43**	3.85	3.28	3.30	**64.87**	**64.55**	**64.69**	3.70
U3M [17]	61.54	2.83	1.71	2.73	62.86	62.23	61.80	2.26
Magic [15]	51.91	0.32	**34.52**	2.66	52.24	52.16	52.57	1.98
AIM-SEEM	33.95	**5.42**	7.04	**28.44**	42.16	41.95	59.92	**11.95**
**Methods**	**AN**	**DN**	**RAD**	**RAN**	**RDN**	**ADN**	**RADN**	**Mean**
CMNeXt [12]	7.58	9.95	50.07	48.77	48.83	8.06	62.76	25.92
MMSFormer [16]	3.57	3.34	**65.00**	65.10	**64.80**	3.40	65.25	36.20
U3M [17]	3.72	2.32	63.35	63.56	62.32	2.62	63.84	34.65
Magic [15]	**36.01**	**37.09**	52.48	52.82	52.73	37.57	64.05	38.74
AIM-SEEM	32.46	31.73	46.57	**70.89**	60.40	**41.22**	**73.12**	**39.15**

**Table 3:** mIoU (%) in the modality-agnostic setting on the DELIVER dataset. R denotes RGB, D denotes Depth, E denotes Event, and L denotes LiDAR. Multi-letter entries indicate modality combinations, such as RD for RGB plus depth and RDEL for using all four modalities. Bold indicates the best, and underline indicates the second-best performance.

**Methods**	**R**	**D**	**E**	**L**	**RD**	**RE**	**RL**	**DE**
CMNeXt [12]	3.76	0.81	1.00	0.72	50.33	13.23	18.22	21.48
MMSFormer [16]	**51.25**	21.24	1.37	4.10	60.52	**50.76**	**50.49**	21.21
U3M [17]	44.23	10.63	0.00	0.23	61.62	43.68	45.40	10.48
Magic [15]	41.97	**57.59**	0.40	0.37	**67.65**	41.93	42.00	**57.62**
AIM-SEEM	47.81	41.29	**9.25**	**9.24**	63.30	48.27	47.91	41.74
**Methods**	**DL**	**EL**	**RDE**	**RDL**	**REL**	**DEL**	**RDEL**	**Mean**
CMNeXt [12]	3.83	2.86	66.24	66.43	15.75	46.29	68.69	23.28
MMSFormer [16]	22.96	3.85	60.28	60.44	**50.01**	22.93	60.23	34.13
U3M [17]	10.77	0.20	61.45	62.43	45.29	11.01	62.26	31.31
Magic [15]	**57.60**	0.27	**67.66**	**67.65**	41.93	**57.63**	72.65	36.93
AIM-SEEM	41.50	**9.25**	65.61	63.08	48.18	41.78	**77.68**	**43.76**

**Table 4:** mIoU/hIoU (%) in the open-vocabulary setting on the MCubes and DELIVER datasets. Base denotes ${\mathrm{mIoU}}_{\mathrm{base}}$, New denotes ${\mathrm{mIoU}}_{\mathrm{new}}$, and H denotes hIoU. Magic [15] results are omitted as their implementation is unavailable. Bold indicates the best result in each column.

**MCubes**										
Methods	K = 1			K = 4			K = 8			Mean
	Base	New	H	Base	New	H	Base	New	H	
CMNeXt [12]	64.85	10.45	18.00	**65.87**	17.25	27.34	67.38	34.52	45.65	30.33
MMSFormer [16]	**65.02**	11.65	19.76	64.54	19.62	30.09	**67.91**	32.21	43.70	31.18
U3M [17]	63.12	9.81	16.98	65.73	16.07	25.83	66.31	28.70	40.06	27.62
AIM-SEEM	63.40	**18.78**	**28.98**	64.59	**26.57**	**37.65**	65.05	**42.96**	**51.75**	**39.46**
**DELIVER**										
Methods	K = 1			K = 4			K = 8			Mean
	Base	New	H	Base	New	H	Base	New	H	
CMNeXt [12]	**75.11**	9.46	16.76	80.57	17.28	28.46	**84.35**	20.39	32.84	26.02
MMSFormer [16]	74.38	10.56	18.49	**81.25**	17.51	28.81	83.74	25.49	39.08	28.79
U3M [17]	70.72	7.48	13.53	73.97	15.32	25.39	80.43	22.49	35.15	24.69
AIM-SEEM	72.80	**14.39**	**24.03**	79.37	**25.07**	**38.10**	82.97	**33.08**	**47.62**	**36.58**

**Table 5:** Component ablation experiment.

Setting	Params (M)	MCubes (%)
RGB fine-tune (baseline)	–	44.32
+Single-scale CMB	+56.2	65.86
+Multi-scale CMB	+98.5	–
+Sum	N/A	N/A
+Average	+0	54.25
+Gated Fusion	+7.22	67.85
+ROA	+0.08	71.09
+VGTT	+1.31	73.13

**Table 6:** Comparison of base-new trade-off strategies under the 8-shot setting.

Setting	Base	New	H
−VGTT	60.38	41.03	48.86
+VGTT (baseline)	65.05	42.96	51.75
+CuPL	64.19	43.15	51.61 (−0.14)
+KgCoOp	63.87	44.08	52.16 (+0.41)
+CoCoOp	64.05	43.98	52.15 (+0.40)

**Table 7:** LLM prompt template for customized prompt generation.

Large Language Model (LLM) Prompts
Describe in detail the color features of the {class}. Just describe it in one sentence.
Describe in detail the texture features of the {class}. Just describe it in one sentence.
Describe in detail the morphological features of the {class}. Just describe it in one sentence.
Describe in detail the physical properties of the {class}, such as hardness, humidity, etc. Just describe it in one sentence.
Describe in detail the optical properties of the {class}, such as reflectivity, glossiness, etc. Just describe it in one sentence.

## Data Availability

The datasets used in this study are publicly available. The MCubes dataset is available at https://github.com/kyotovision-public/multimodal-material-segmentation (accessed on 19 January 2026), and the DELIVER dataset is available at https://github.com/InSAI-Lab/DELIVER (accessed on 19 January 2026). The code is available at https://github.com/ExcellantQian/SEEM-for-Arbitrary-Imaging-Modalities (accessed on 19 January 2026).

## Abbreviations

The following abbreviations are used in this manuscript:

AIM	Arbitrary Imaging Modalities
AIM-SEEM	SEEM for Arbitrary Imaging Modalities
SEEM	Segment Everything Everywhere All at Once
ROA	RGB-Oriented Aligner
CMB	Cross-Modal Blender
VGTT	Visual-Guided Text Tuner
AMSS	Arbitrary Modality Semantic Segmentation
OVS	Open-Vocabulary Segmentation
PCW	Pyramid Channel Weighting
RGB	Red, Green, and Blue
NIR	Near-Infrared
DoLP	Degree of Linear Polarization
AoLP	Angle of Linear Polarization
LiDAR	Light Detection and Ranging
IoU	Intersection over Union
mIoU	Mean Intersection over Union
hIoU	Harmonic Mean of Base-Class and New-Class mIoU
CLIP	Contrastive Language-Image Pre-training
SAM	Segment Anything Model
LLM	Large Language Model
